# Use of a Large Language Model to Reveal Narrative Architectures of Veteran Transition Stress: Development and Validation Study

**DOI:** 10.2196/90155

**Published:** 2026-04-30

**Authors:** Isaac R Galatzer-Levy, Xi Pan, Roland P Hart, George A Bonanno

**Affiliations:** 1 Google DeepMind, New York, NY, United States; 2 Department of Clinical Psychology, Teachers College, Columbia University, 525 West 120th Street, Box 303, New York, NY, 10027, United States, 1 2126783468

**Keywords:** large language models, veteran mental health, transition stress, natural language processing, thematic analysis, psychological assessment

## Abstract

**Background:**

The stress caused by multiple aspects of veterans’ transitions from military to civilian, termed transition stress, represents a unique source of psychological impact that is underresearched due to its qualitative nature. The assessment of this complex psychological phenomena has thus relied on laborious interviews designed to extract quantitative information from qualitative narratives of the transition to civilian life. We sought to determine if large language models (LLMs) could be used as valid measurement tools to extract relevant information from open-ended narratives.

**Objective:**

This study sought to develop and validate a generative artificial intelligence (AI) approach to automate the quantification and subsequent thematic analysis of veteran transition stress.

**Methods:**

Utilizing transcripts from interviews of a sample of US military veterans, we developed an LLM to rate transition stress severity and examined the model’s reliability in relation to human coders and validity in relation to a set of related questionnaire measures. Next, we used the LLM scores to quantitatively define high and low transition stress groups, enabling a targeted, automated analysis of themes related to narrative identity and life transition themes that might differentiate the two groups.

**Results:**

LLM ratings of transition stress correlated highly with the human expert ratings and showed significant, theoretically congruent correlations with measures of clinical symptoms, reintegration difficulties, and veterans’ self-ratings of transition difficulty. Critically, the AI-derived thematic analyses of the narratives from high and low transition stress veterans revealed clearly distinct and informative patterns.

**Conclusions:**

These findings suggest that generative AI offers a robust, scalable, and reliable method for multidimensional analysis of complex, narrative-based psychological constructs.

## Introduction

The transition from military service to civilian life is a period of profound adjustment for veterans, frequently marked by significant stress distinct from posttraumatic stress disorder (PTSD) and other service-related pathology [1]. This “transition stress” encompasses a wide array of challenges, including adapting to civilian employment [2,3], re-establishing interpersonal relationships [4], navigating a less structured lifestyle, and finding a new sense of purpose and identity [3,5,6]. Although a minority of veterans experience PTSD, a substantial proportion encounters high levels of transition-related stress [1], which can predict subsequent mental and physical health problems and increase vulnerability to outcomes, such as suicide [7-9]. The period immediately following separation can represent a “deadly gap” where veterans may lose access to military support systems before connecting with civilian or Veterans Administration (VA) services, exacerbating stress and risk [9].

Transition stress is multidimensional, posing a challenge for traditional measurement approaches and making it difficult to identify what differentiates successful from unsuccessful transitions. Traditional self-report questionnaires, while scalable, may not fully capture these complex, dynamic experiences, blurring their distinction from other measures of distress or psychopathology and rendering them susceptible to recall bias [10]. Qualitative methods, such as in-depth interviews like the loss, trauma, and transition stress (LOTTS) interview [11], provide rich, nuanced data essential for understanding these idiographic experiences. However, analyzing such data typically requires extensive human expert labor, limiting scalability and introducing potential rater variability.

In this study, we sought to address these challenges by applying generative artificial intelligence (AI)—systems that can generate and interpret text—to quantify veteran transition stress from open-ended narrative data. Large language models (LLMs), a class of generative AI, can process complex narratives and identify subtle patterns relevant to psychological constructs, offering transformative potential for psychological measurement. This study represents an early empirical application of generative psychometrics—a framework that uses LLMs to derive quantitative psychological measurements from unstructured text [12,13]—to develop and validate an LLM (Gemini 2.5 Flash) for assessing veteran transition stress. Our study was guided by 2 primary aims. First, we sought to evaluate the interrater reliability and construct validity of AI-generated transition stress ratings against human experts and established clinical measures. We hypothesized (hypothesis 1) high agreement and significant, positive correlations with measures of psychological distress and reintegration difficulties.

Second, we tested a novel methodological application wherein the AI-generated quantitative scores were used to define distinct groups (eg, high vs low stress) to facilitate a targeted, automated qualitative analysis of narrative themes relevant to identity and life transition [1,3,14], specifically, personal agency, communion, identity continuity, stereotype threat, redemption/contamination arc, coping, and challenge framing. Together, these thematic dimensions constitute what we refer to as the “narrative architecture” of transition stress—that is, the overarching structural patterns through which veterans organize and convey their transition experiences. We hypothesized (hypothesis 2) that this approach would reveal clear and divergent thematic patterns.

## Method

### Ethical Considerations

All procedures were approved by the Teachers College, Columbia University Institutional Review Board (protocol number: 21-104). Participants provided informed consent prior to participation and provided additional verbal consent immediately before the interview, at which time they were informed that their interview transcripts could be analyzed using machine-learning methods.

Consent materials specified that to maintain participants’ confidentiality and anonymity, they would be assigned a code number, that the association of the code number with their name would be kept in a password-protected file and stored on a secured server, that the code number would be used to record participants’ responses to all tasks and questionnaires and that their name would never be recorded on any task or questionnaire data, that only the principal investigator and members of the research team would view participants’ written, electronic, or digital materials, and that these materials would be stored on a password-protected computer. Consent materials also informed participants that the investigators may wish to contact them in the future regarding this study or a possible future study and asked them to initial a statement on the consent form indicating whether they were willing or not willing to be contacted for these purposes in the future. Participants were paid US $60 for completion of the questionnaires and interview.

### Participants

Participants were 106 US military veterans recruited via online social media platforms (eg, Reddit) with permission from veteran-specific forum moderators and from prior related studies in which participants had consented to be recontracted for future research as part of a larger veteran project. Eligibility criteria included being 18 to 70 years of age, residing in the United States, and having served in the military after September 11, 2001. Veteran status was verified prior to participation through official documentation (DD214, military ID card, or VA ID card). Demographic characteristics are presented in Table 1.

**Table 1.** Participant demographic and clinical characteristics^a^.

Characteristics	Women (n=20)	Men (n=86)	Total (N=106)
Age (years), mean (SD)	31.15 (6.73)	36.81 (8.65)	35.75 (8.58)
Military AD years, mean (SD)	6.37 (3.82)	12.89 (8.73)	11.68 (8.43)
Military AD end year, mean (SD)	2019.50 (3.63)	2017.58 (5.11)	2017.94 (4.90)
Hispanic, n (%)			
No	18 (90)	76 (88.4)	94 (88.7)
Yes	2 (10)	10 (11.6)	12 (11.3)
Race/ethnicity, n (%)			
American Indian/Alaska Native	0 (0)	7 (8.1)	7 (6.6)
Asian	1 (5)	8 (9.3)	9 (8.5)
Black/African American	5 (25)	0 (0)	5 (4.7)
Other/multiracial	1 (5)	11 (12.8)	12 (11.3)
Prefer not to answer	1 (5)	1 (1.2)	2 (1.9)
White	12 (60)	61 (70.9)	73 (68.9)
Education, n (%)			
High school or equivalent	3 (15)	17 (19.8)	20 (18.9)
Bachelor’s degree	7 (35)	27 (31.4)	34 (32.1)
Master’s/graduate degree	6 (30)	30 (34.9)	36 (34)
Other	4 (20)	12 (14)	16 (15.1)
Marital status, n (%)			
Married	11 (55)	54 (62.8)	65 (61.3)
Single	6 (30)	20 (23.3)	26 (24.5)
Divorced	2 (10)	12 (14)	14 (13.2)
Domestic partnership	1 (5)	0 (0)	1 (0.9)
Household income (US $), n (%)			
<25,000	6 (30)	7 (8.1)	13 (12.3)
25,000-40,000	4 (20)	13 (15.1)	17 (16)
40,000-60,000	2 (10)	10 (11.6)	12 (11.3)
60,000-80,000	4 (20)	9 (10.5)	13 (12.3)
80,000-100,000	2 (10)	11 (12.8)	13 (12.3)
>100,000	2 (10)	36 (41.9)	38 (35.8)
Military branch, n (%)			
Army	13 (65)	56 (65.1)	69 (65.1)
Air force	3 (15)	4 (4.7)	7 (6.6)
Marine corps	0 (0)	11 (12.8)	11 (10.4)
Navy	1 (5)	10 (11.6)	11 (10.4)
Multiple	3 (15)	5 (5.8)	8 (7.5)
Deployed, n (%)			
Yes	11 (55)	70 (81.4)	81 (76.4)
No	9 (45)	16 (18.6)	25 (23.6)

a Values for continuous variables are presented as mean (SD); categorical variables are presented as n (%), with percentages calculated within gender. Race/ethnicity categories were not mutually exclusive; participants could endorse more than 1 category; therefore, percentages may sum to greater than 100%. Interviews were conducted between 2021 and 2023.

### Procedure

Following consent, participants completed a semistructured interview assessing military-to-civilian transition experiences using the LOTTS interview protocol [11]. All interviews were conducted remotely via Zoom by graduate-level research assistants who received standardized training in the LOTTS protocol and were required to meet competency criteria established prior to conducting interviews independently.

Interviews were semistructured and included standardized prompts with follow-up questions used to clarify and elaborate on participants’ responses as needed. Total interview duration ranged from approximately 20 to 40 minutes, depending on the number and type of experiences reported (eg, losses and deployment experiences). The transition narrative segment used for the present analyses consisted of an open-ended prompt in which participants were invited to describe their transition to civilian life for up to 7 minutes. All interviews were transcribed and deidentified prior to analysis. Participants also completed standardized self-report clinical measures.

**Figure 1.**
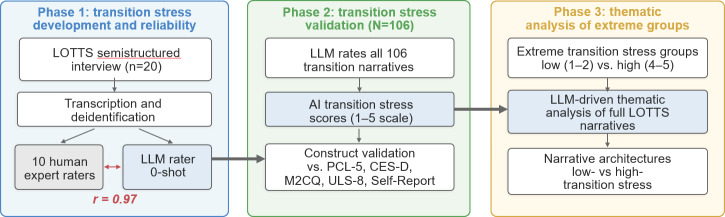
Conceptual overview of the generative psychometrics pipeline. AI: artificial intelligence; CES-D: Center for Epidemiologic Studies Depression Scale; LLM: large language model; LOTTS: loss, trauma, and transition stress; M2CQ: Military to Civilian Questionnaire; PCL-5: PTSD Checklist for DSM-5; ULS-8: UCLA Loneliness Scale, 8-item version.

A multiphase approach was used (see Figure 1). In phase 1, the senior author (GAB) convened repeated meetings with 10 experts who had prior experience working with veteran populations. These iterative meetings served as the training and calibration procedure: the team collaboratively developed the operational definition of veteran transition stress and the 5-point rating scale with descriptive anchors at each level, ensuring a shared understanding of the construct and rating criteria before independent coding began. The 10 experts then independently rated 20 randomly selected transcripts for transition stress based on the consensus criteria (see Materials in MultimediaAppendices 1  2). Raters were provided only with deidentified transition narrative transcripts and the rating criteria; they did not have access to participants’ clinical measure scores, demographic information, or other raters’ scores during the coding process. Individual rater disagreements were not adjudicated through consensus discussion. Instead, the average of all 10 expert ratings was used as the composite human score for each transcript, preserving natural rater variability essential for a meaningful assessment of interrater reliability. These composite scores were then compared to ratings from an LLM for transition stress modeled after the same consensus criteria.

All AI ratings were generated using Google’s Gemini 2.5 Flash model, accessed via the Google Gemini application programming interface (API). The model version was locked for the duration of the study to ensure consistency across all rating sessions. The following API parameters were used: temperature=0 (greedy decoding, to minimize stochastic variation across runs), and max output tokens were set to accommodate both a numerical rating and a written explanation for each transcript. No system-level instructions were used beyond the prompt itself. The prompt structure for the transition stress rating task consisted of 3 components. First, a role assignment instructed the model to adopt the perspective of a veteran transition stress rater (“You are a veteran transition stress rater”). Second, the complete operational definition of transition stress was provided verbatim within the prompt, specifying that transition stress refers to the psychological and emotional strain experienced during periods of significant change for veterans, including domains such as employment, family reacclimation, relationships, finances, and schedule management. Third, the full 5-point rating scale with anchored descriptors (1=minimal or no distress; 2=minimal to moderate; 3=moderate; 4=moderate to high; and 5=high distress) was included. The model was then instructed to rate the level of transition stress in each attached transcript according to the definition and scale and to provide explanations for each rating.

Five prompting conditions were tested to evaluate the effect of in-context learning on rating performance: 0-shot (definition and scale only, no examples), 1-shot (1 expert-rated example transcript), 2-shot (2 examples), 3-shot (3 examples), and 5-shot (5 examples). In the few-shot conditions, example transcripts were selected to span the full range of the rating scale and were accompanied by the expert consensus rating (mean of 10 raters) and 3 illustrative expert explanations that articulated the rationale for the assigned rating. Specifically, the 1-shot condition included a high-stress example (expert mean 4.9/5, SD 0.32); the 2-shot condition added a low-stress example (expert mean 1.0/5, SD 0); the 3-shot condition added a moderate-stress example (expert mean 3.8/5, SD 0.42); and the 5-shot condition further added a low-moderate example (expert mean 2.0/5, SD 0.82) and a moderate example (expert mean 3.0/5, SD 0.67). Example transcripts were excluded from the rating set for their respective conditions to prevent circularity (eg, in the 1-shot condition, 19 of 20 calibration transcripts were rated; in the 5-shot condition, 15 were rated). Transcripts were provided to the model as attached Excel files containing the raw narrative text from the LOTTS transition interview. The complete prompts for all 5 conditions are provided in Multimedia Appendix 3.

The 0-shot condition was selected as the primary analysis because it achieved the highest agreement with human experts (intraclass correlation coefficient [ICC](A,k)=0.954, *r*=0.955) while requiring no training examples, maximizing scalability and eliminating any risk of overfitting to the calibration sample. The 0-shot prompt was subsequently applied to all 106 transcripts in the full sample for the construct validity and thematic analyses reported in Phases 2 and 3.

### Materials

#### Clinical Self-Report Measures

A battery of validated clinical measures was administered that included the PTSD checklist for *DSM-5* (*Diagnostic and Statistical Manual of Mental Disorders*, Fifth Edition) [15], Center for Epidemiologic Studies Depression Scale [16], the State-Trait Anxiety Inventory [17], Military to Civilian Questionnaire (M2CQ) [18] as a measure of reintegration difficulties, and UCLA Loneliness Scale-8 [19].

#### LOTTS Interview

The LOTTS [11] is a semistructured interview that queries respondents about both residual stressors from deployment-related loss and trauma experiences and ongoing stressors and rewards from the transition to civilian life. To address the limitations of conventional self-reports, the LOTTS combines free-response interview questions with follow-up structured self-report items. This format has been shown to more thoroughly probe respondents’ memory, minimize response bias, and clarify and focus answers [10]. The deployment-related portion of the LOTTS included respondents’ overall experience of deployment as well as specific experiences pertaining to the death of close friends or other meaningful relationships, being seriously injured, witnessing others being seriously injured, or witnessing or participating in the killing of others. The transition-related portion of the LOTTS included respondents’ overall experience of the transition as well as specific experiences pertaining to civilian employment; finances; interpersonal relationships at work, with spouse and children, with other family, and with nonmilitary friends; managing leisure time; and adjusting to civilian laws.

#### Transition Stress Ratings

To code transition stress, we used the free-response portion of the LOTTS pertaining to respondents’ overall experience of the transition. For this portion, participants were given up to 6 minutes to respond orally to the following prompt.

*I would like you to describe what the transition from active duty to civilian life has been like for you. What has been especially difficult in the transition? What has been rewarding? We’d like to hear about any aspects of the transition that might have been rewarding or difficult for you. This could pertain to family life, friends, finding work, or housing, or how you spend your time, or your general feelings about leaving the military or about civilian life.*

Immediately following this segment, participants were asked to rate how “difficult/demanding” their transition experience had been.

Transition stress from participants’ narrative responses was operationally defined as

*The psychological and emotional strain experienced during periods of significant change or transition in life. For veterans, these changes may include moving to a new location, finding work and adjusting to civilian employment, reacclimating life with their spouse, children or other relatives, relationships with non-military friends, managing finances, and managing civilian time and schedule. Transition stress arises from the uncertainty, adjustment, and adaptation required to navigate these changes successfully.*

Ratings used a 5-point ordinal scale (1=minimal distress to 5=high distress). Ten human expert raters coded transcripts independently. The AI rater utilized Gemini 2.5 Flash (Google AI Studio) in a 0-shot condition. The LLM was provided with the full operational definition of veteran transition stress and the complete 5-point ordinal rating scale with descriptive anchors at each level (1 = “minimal or no distress from transition-related challenges” through 5 = “high distress due to transition-related challenges”). No calibration examples, training transcripts, or illustrative rationales were provided in the 0-shot condition. We also explored 1-, 2-, 3-, and 5-shot conditions, accompanied by human expert consensus ratings and illustrative rationales. However, because the 0-shot condition achieved extremely high reliability with human coders (see Results), these additional conditions did not increase reliability and were not further considered in the analyses.

### Data Analysis

#### Quantitative Validation

Interrater reliability among human experts was assessed using multiple complementary indices: intraclass correlation coefficients for average measures (ICC [A,k]) and single measures (ICC [A,1]), both reflecting absolute agreement in a 2-way random-effects model, as well as Krippendorff α for ordinal data. AI-human agreement was evaluated using ICC (A,k), Pearson correlation with 95% CIs, mean absolute error, and root mean square error. Construct validity was assessed through both convergent and discriminant validity evidence. Convergent validity was evaluated using Pearson correlations between AI-rated transition stress and clinical measures of psychological distress and reintegration difficulties. A comprehensive correlation matrix, including all study variables, descriptive statistics, and pairwise sample sizes, is presented in Table 2. Four primary hypothesized correlations (PTSD symptoms, depression, loneliness, and reintegration difficulties) were specified a priori. To address multiple comparisons, we applied Benjamini-Hochberg false discovery rate (FDR) correction. Partial correlations controlling for age and gender were also computed. Discriminant validity was assessed by correlating AI ratings with surface-level linguistic features of the transcripts—including word count, sentence count, mean words per sentence, type-token ratio, and mean word length—to confirm that the AI was rating psychological content rather than narrative verbosity or linguistic complexity. Post hoc power analyses were conducted using the pwr package in R software (R Foundation)[20]. With n=106 and α=.05, the study was powered at 0.95 to detect correlations of *r*≥0.34 and at 0.88 to detect *r*≥0.30.

**Table 2.** Intercorrelations for AI-rated transition stress and clinical measures.

Variable	[1]	[2]	[3]	[4]	[5]	[6]	[7]	[8]
AI transition stress^a^	—^b^							
Self-rated difficulty^c^	0.70^d^	—						
PTSD^e^ (PCL-5^f^)	0.40^d^	0.41^d^	—					
Depression (CES-D^g^)	0.34^d^	0.42^d^	0.74^d^	—				
Trait anxiety (STAI-T^h^)	0.25^j^	0.34^k^	0.68^d^	0.81^d^	—			
State anxiety (STAI-S^i^)	0.22^j^	0.32^k^	0.61^d^	0.79^d^	0.92^d^	—		
Loneliness (ULS-8^l^)	0.42^k^	0.37^j^	0.34^j^	0.51^d^	0.49^k^	0.38^j^	—	
Reintegration (M2CQ^m^)	0.38^d^	0.39^d^	0.75^d^	0.83^d^	0.82^d^	0.80^d^	0.47^k^	—

a AI transition stress=Gemini 2.5 Flash 0-shot rating (1‐5 scale).

b Not applicable.

c Participant self-report of transition difficulty from the LOTTS interview (Trans_10).

d *P*<.001.

e PTSD: posttraumatic stress disorder.

f PCL-5: PTSD checklist for DSM-5.

g CES-D: Center for Epidemiologic Studies Depression Scale.

h STAI-T: State-Trait Anxiety Inventory, Trait subscales.

i STAI-S: State-Trait Anxiety Inventory, State subscales.

j *P*<.05.

k *P*<.01.

l ULS-8: UCLA Loneliness Scale, 8-item version.

m M2CQ: Military to Civilian Questionnaire.

#### Qualitative Thematic Analysis

Following the quantitative rating, participants were divided into a low transition stress group (AI scores 1‐2) and a high transition stress group (AI score 4‐5). The LLM was then prompted in a separate analytical step to act as a qualitative researcher, extracting and summarizing the dominant narrative themes from the entire LOTTS interview that differentiated these 2 quantitatively defined groups. The analyses were guided by targeted themes from the identity and transition stress literature, specifically personal agency, communion, identity continuity, stereotype threat, redemption/contamination arc, coping, and challenge framing. We also analyzed themes that were particularly salient for different demographic groups, such as gender. This demonstrates a method for using quantitatively scored narratives to guide deeper thematic exploration of other psychological processes within the same qualitative data. The threshold for defining extreme groups (low: scores 1‐2; high: scores 4‐5) was selected to capture participants at the clearly distinct ends of the transition stress continuum while excluding the ambiguous middle category (score 3; n=26, 24.5% of the sample). This yielded a low transition stress group of 37 (34.9%) and a high transition stress group of 43 (40.6%). To evaluate the robustness of this grouping, sensitivity analyses were conducted using 5 alternative threshold definitions: (a) low 1‐2 versus high 3‐5, (b) low 1‐3 versus high 4‐5, (c) extreme groups only (score 1 vs score 5), (d) median split, and (e) tertile split. Group differences on key clinical measures (PTSD, depression, loneliness, and reintegration difficulties) were compared across all threshold schemes using independent-samples *t* tests and Cohen *d*.

## Results

### Quantitative Validation of AI Ratings

Ten human experts rated 20 randomly selected transcripts. The average-measures ICC for absolute agreement was excellent (ICC(A, 10)=0.978, 95% CI 0.960-0.990; *F*_(19, 160)_=49.77, *P*<.001). The single-measures ICC was also strong (ICC(A, 1)=.817, 95% CI 0.708-0.908), indicating that even individual raters achieved good reliability. Krippendorff α for ordinal data was α=0.799, approaching the conventional threshold of 0.80. Pairwise correlations among the 10 raters ranged from 0.61 to 0.97 (mean 0.83, SD 0.10). Individual rater means ranged from 2.25 to 2.95 (on the 1‐5 scale), indicating minimal systematic rater bias. The zero-shot AI rater (Gemini 2.5 Flash) achieved excellent agreement with the human expert mean: ICC(A, k) 0.954, 95% CI 0.823-0.984; *r*=0.955, 95% CI 0.887-0.982; *P*<.001; mean absolute error=0.49; root mean square error=0.61. Performance was comparable to or slightly lower in few-shot conditions (see Table 3), confirming that additional training examples did not improve upon 0-shot performance. To evaluate whether exemplar-based calibration would improve scale interpretation, we additionally tested 1-, 2-, 3-, and 5-shot prompting conditions. In these conditions, the model was provided with human expert consensus ratings and qualitative rationales for representative transcripts spanning the rating scale. The 0-shot condition matched or exceeded the reliability of all few-shot conditions, suggesting that the construct definition and scale descriptors were sufficient for the model to achieve consistent interpretation aligned with human expert judgment.

**Table 3.** AI-human agreement across prompting conditions^a^.

Condition	n	ICC(A, k)^b^ (95% CI)	*r* (95% CI)	MAE^c^	RMSE^d^
0-shot	20	0.954 (0.823-0.984)	0.955 (0.887-0.982)	0.49	0.61
1-shot	19	0.957 (0.887-0.983)	0.963 (0.905-0.986)	0.45	0.58
2-shot	18	0.949 (0.713-0.985)	0.952 (0.874-0.982)	0.46	0.58
3-shot	17	0.867 (0.228-0.963)	0.862 (0.651-0.949)	0.68	0.88
5-shot	15	0.899 (0.170-0.975)	0.914 (0.755-0.971)	0.68	0.84

a Agreement between AI rater (Gemini 2.5 Flash) and Human Expert Mean Across Prompting Conditions (n=20 transcripts). Sample sizes vary due to occasional AI parsing failures in few-shot conditions.

b ICC(A, k): intraclass correlation coefficient, 2-way random, absolute agreement, average measures.

c MAE: mean absolute error.

d RMSE: root mean square error.

As hypothesized, AI-rated transition stress showed significant positive correlations with clinical measures after FDR correction: PTSD symptoms (*r*=0.40, p_FDR<.001), depression (*r*=0.34, p_FDR=0.001), loneliness (*r*=0.42, p_FDR=0.011), and reintegration difficulties (*r*=0.38, p_FDR <0.001). These associations were robust to demographic controls; partial correlations controlling for age and gender were virtually unchanged (partial r range: 0.21–0.43; see Table 4). The AI-rated transition stress also correlated significantly with trait anxiety (*r*=0.25, p_FDR=0.042). AI-rated transition stress also showed a strong positive correlation with participants’ self-ratings of experiencing a “difficult/demanding” transition (*r*=0.70, *P*<.001). Regressing both the AI-rated transition stress and questionnaire-based reintegration difficulties (M2CQ) onto participant self-ratings explained more than half the variance (*R*²=0.51, *F*_(2, 98)_=50.43, *P*<.001), with the AI-rated score emerging as the more robust predictor (*β*=.72, *P*<.001, sr²=.60) compared to the M2CQ (*β*=.01, *P*=.06, sr²=.13).

To rule out the possibility that AI ratings were driven by surface-level text properties rather than psychological content, we correlated transition stress scores with 5 linguistic features of the transcripts (Table 5). Linguistic complexity indicators—type-token ratio (*r*=.05, *P*=.59), mean word length (*r*=0.14, *P*=.16), and mean words per sentence (*r*=−0.11, *P*=.29)—were not significantly associated with AI ratings, confirming that the model was not simply responding to vocabulary sophistication or syntactic complexity. Narrative length (word count: *r*=0.32; sentence count: *r*=0.24) showed modest positive correlations with AI scores, which is expected and substantively interpretable: veterans experiencing greater distress had more to report about their transition difficulties. Crucially, this association is content-driven—more distress generates more narrative content—rather than an artifact of the AI rewarding verbosity per se.

**Table 4.** Convergent validity with multiple comparisons correction^a^.

Measure	n	*r* (95% CI)	*P* value^b^	*P*_FDR^c^	Partial *r*^d^
PTSD^e^ symptoms (PCL-5^f^)	103	0.397 (0.220-0.548)	<.001	<.001^h^	0.401
Depression (CES-D^g^)	104	0.342 (0.160-0.502)	<.001	.001^h^	0.343
Loneliness (ULS-8^i^)	45	0.420 (0.145-0.635)	.004	.01^h^	0.429
Reintegration (M2CQ^j^)	101	0.382 (0.202-0.538)	<.001	<.001^h^	0.384
Trait anxiety (STAI-T^k^)	85	0.246 (0.035-0.436)	.02	.04^h^	0.244
State anxiety (STAI-S^l^)	96	0.217 (0.018-0.400)	.03	.05	0.214

a Note: Correlations between AI-rated transition stress and clinical measures (n=106).

b *P*=uncorrected *P *value.

c p_FDR=Benjamini-Hochberg false discovery rate.

d Partial *r*=controlling for age and gender.

e PTSD: posttraumatic stress disorder.

f PCL-5: PTSD Checklist for DSM-5.

g CES-D: Center for Epidemiologic Studies Depression Scale.

h Significance at α=.05 after the respective correction.

i ULS-8: UCLA Loneliness Scale, 8-item version.

j M2CQ: Military to Civilian Questionnaire.

k STAI-T: State-Trait Anxiety Inventory, Trait subscales.

l STAI-S: State-Trait Anxiety Inventory, State subscales.

**Table 5.** Discriminant validity—correlations between artificial intelligence (AI)–rated transition stress and linguistic feature^a^.

Linguistic feature	*r* (95% CI)	*P* value
Complexity indicators		
Type-token ratio	0.054 (−0.139 to 0.242)	.59
Mean word length	0.136 (−0.056 to 0.318)	.17
Mean words per sentence	−0.105 (−0.290 to 0.087)	.28
Length indicators		
Word count	0.321 (0.139 to 0.482)	<.001
Log word count	0.352 (0.173 to 0.509)	<.001
Sentence count	0.236 (0.047 to 0.408)	.02

a Modest correlations between narrative length and AI ratings are substantively expected: veterans with greater transition stress had more to report about their difficulties. Critically, linguistic complexity indicators (type-token ratio, mean word length, and mean words per sentence), which would indicate the AI responding to vocabulary sophistication or syntactic structure rather than content, were uniformly nonsignificant.

### Qualitative Thematic Analyses of Extreme Transition Stress Groups

AI transition stress score distribution across the 106 transcripts was as follows: score 1: 14 (13.2%); score 2: 23 (21.7%); score 3: 26 (24.5%); score 4: 17 (16.0%); score 5: 26 (24.5%). The primary grouping yielded 37 low (scores 1‐2) and 43 high (scores 4‐5), with 26 participants (24.5%) excluded at score 3. Sensitivity analyses confirmed that high versus low group differences on all 4 primary clinical measures were robust across all 6 threshold definitions tested, with Cohen *d* values consistently in the medium-to-large range (*d*=0.57‐2.01 across schemes; see Table 6). The most extreme contrast (score 1 vs 5) yielded the largest effects (*d*=0.87‐2.01), while broader groupings (eg, median split) produced attenuated but still significant effects (*d*=0.74‐0.81), confirming that the primary threshold captures meaningful group differences without being dependent on a specific cutoff.

**Table 6.** Sensitivity analysis: group differences across alternative threshold definitions^a^.

Threshold scheme	n_Low	n_High	n_Excluded	PTSD^b^ *d^c^*	Depression *d^c^*	Loneliness *d^c^*	M2CQ *d^c^*
Primary: 1‐2 versus 4‐5	37	43	26	0.86^f^	0.81^f^	1.13^e^	0.89^f^
Low 1‐2 versus high 3‐5	37	69	0	0.57^e^	0.56^e^	0.87^e^	0.70^e^
Low 1‐3 versus high 4‐5	63	43	0	0.81^f^	0.74^f^	0.76^d^	0.76^f^
Extreme: 1 versus 5	14	26	66	1.23^f^	0.87^d^	2.01^e^	1.00^e^
Median split	63	43	0	0.81^f^	0.74^f^	0.76^d^	0.76^f^
Tertile split	37	43	26	0.86^f^	0.81^f^	1.13^e^	0.89^f^

a Significance from independent-samples *t* tests. All threshold schemes produce significant group differences in the same direction, confirming robustness of the primary grouping.

b PTSD: posttraumatic stress disorder.

c *d*=Cohen *d* (high to low).

d *P*<.05.

e *P*<.01.

f *P*<.001.

A qualitative thematic analysis comparing low and high transition stress groups, as defined by the AI’s scores, along transition stress themes revealed markedly distinct narrative patterns. The narrative arc of veterans in the low transition stress group was redemptive and coherent and followed a logical cause-and-effect structure focused on starting a new chapter in the present and future. These narratives described themes of high agency (proactive mastery) and communion (building new sources of support), a continuity of identity (military experience integrated as foundation part of self), and low stereotype threat (military service is viewed as one part of a broader self-concept). Coping was described as planful, and problem-focused coping and challenges were framed as manageable “bumps in the road.” In stark contrast, the narrative arc from veterans in the high transition stress group was one of contamination and fragmentation. These narratives articulated themes of low agency (passive victim) and communion (profound isolation), a fractured identity (profound grief over loss of military self), and high stereotype threat (hyperawareness of negative labels and pervasive sense of being misunderstood by civilians). Coping was described as reactive and ineffective, and challenges were framed as insurmountable, leading to spirals of despair. More details on these divergent narrative themes are presented in Table 7. Illustrative text examples of 1 thematic component, narrative arc, along with the AI explanation for the categorization of that component are given in Table A1 in Multimedia Appendix 1.

**Table 7.** Key narrative themes of low and high transition stress groups.

Theme	Low transition stress	High transition stress
Agency	Internal (proactive mastery): Transition is narrated as a strategic maneuver. The veteran is the active protagonist driving the plot.	External (passive victim): Transition is forced by external factors (medical/legal). The veteran feels acted upon by the system.
Communion	Support rebuilding: Successful shift of “tribe” from unit to family, school cohorts, or new civilian communities.	Profound isolation: Loss of the military “brotherhood” without replacement; fear of being seen as “broken” or misunderstood by civilians.
Identity continuity	Identity integration: Military service is integrated as a foundational part of the self, but not the whole self.	Loss of military self: Profound grief; the veteran cannot separate their worth from their rank/role, leading to an existential void.
Stereotype threat	Integrated identity: Military service is viewed as just a part of a broader self-concept.	Broken warrior: Hyperawareness of negative labels and fear of becoming a “statistic.” Pervasive sense of being misunderstood or viewed as a “pariah” by civilians.
Narrative arc	Redemptive and coherent: Stories follow a logical cause-and-effect structure. The narrative arc is coherent and redemptive with a focus on starting a new chapter in the present and future.	Contaminated and fragmented: Narratives are characterized by a cascade of crises and are often anchored in past trauma. The story arc is defined by an event so powerful it “contaminates” all other aspects of life.
Coping	Proactive: Coping is problem-focused and planful.	Ineffective: Coping is reactive and maladaptive, heavy alcohol use or full shutdown.
Challenge framing	Manageable: Challenges are framed as “bumps in the road”; Bureaucracy and cultural differences as annoyances to be navigated.	Functional collapse: Bureaucracy and cultural differences feel insurmountable; obstacles lead to spirals of despair.

### Qualitative Thematic Analyses by Gender

A meaningful gender disparity also emerged. While the sample of female veterans was small (n=20), they were disproportionately represented in the high transition stress group. Their narratives frequently centered on military sexual trauma coupled with a profound sense of institutional betrayal by leadership, which together became the defining, unresolved theme that precipitated a severely difficult transition (see Table A2 in Multimedia Appendix 2).

One female veteran encapsulated this theme of compounded trauma:

*It’s been pretty horrible. I got out because of military sexual trauma... I basically agreed with my commander and helped them cover up what happened to me as long as they let me out honorably and gave me my benefits, and I feel that, that regret to this day.*

[AI Transition Stress Score=5]

## Discussion

### Theoretical Implications: The Dual Pathways of Transition Stress

The findings of this study offer a nuanced, dual-pathway understanding of veteran transition stress, revealing that successful and difficult transitions are not merely quantitatively different but are defined by fundamentally distinct narrative structures. A primary insight is that the simple presence of potentially traumatic events during service, such as combat exposure or the loss of comrades, does not predict a high-stress transition by itself. Rather, the crucial differentiator appears in how these experiences are processed, integrated, and framed within the veteran’s transition story. The narratives of low transition stress veterans were characterized by a sense of agency and proactive, problem-focused coping, often involving detailed pretransition planning and the leveraging of social support systems. While many of these individuals experienced significant trauma, their narratives contextualized these events as difficult past experiences that had been processed or compartmentalized, allowing for a future-oriented mindset focused on civilian opportunities. Their stories follow a classic “redemptive” arc—a problem-solution structure where initial hardships are reframed as necessary steps toward a better life.

Conversely, the narratives of high transition stress veterans were defined by compounding crises and a pervasive lack of agency and control. Their transition from the military often occurred involuntarily or unexpectedly, precipitated by medical retirement or personal trauma, and was frequently followed by a cascade of stressors, including unemployment and relationship breakdown. For this group, past traumatic experiences remain a central feature of their present-day experience, fueling a narrative of identity loss, profound social isolation, and being psychologically “stuck” in the past. These stories followed a narrative arc of contamination and fragmentation, dominated by events so powerful they psychologically tainted all other aspects of the veteran’s life. Furthermore, these analyses uncovered a critical gender dimension, where female veterans were disproportionately represented in the high transition stress group. Their narratives were uniquely characterized by military sexual trauma and institutional betrayal, suggesting that the source of stress for these veterans is often the pain of unresolved trauma perpetrated and then inadequately addressed by the institution they served.

### Methodological Advances: Generative Psychometrics

These substantive insights were garnered through the application of a novel generative psychometrics framework, which this study served to validate by first establishing the quantitative robustness of AI-driven measurement. Operating in a 0-shot condition, the LLM achieved excellent interrater reliability with 10 human clinical experts (*r*=0.97). The construct validity of the AI’s ratings was further supported by theoretically congruent correlations with validated clinical measures of PTSD, depression, and loneliness, as well as a strong correlation (*r*=0.70) with the veterans’ own self-ratings of transition difficulty.

This framework provides a significant qualitative advance over prior automated text analysis methods in psychology. Traditional dictionary-based tools, such as linguistic inquiry and word count [21], quantify predetermined linguistic categories but cannot account for context, narrative structure, or semantic meaning—a limitation that obscures the clinically meaningful complexity of simultaneous relief and grief or the difference between effectively managed anxiety and debilitating distress. Similarly, while topic-modeling approaches (eg, Latent Dirichlet Allocation) [22] identify latent thematic clusters, they are unsupervised and atheoretical, failing to produce participant-level severity ratings anchored to external criteria. The present LLM-based approach overcomes these limitations by combining emergent thematic pattern recognition with the generation of construct-specific, ordinally scaled ratings. By processing meaning in context, the LLM approximates the integrative judgment historically requiring trained human raters.

This approach also represents a paradigm shift regarding scalability. While the human expert panel required an estimated 175 to 265 person-hours to evaluate the 106-transcript corpus, the AI completed the task in less than 30 minutes of computation time. However, this efficiency does not eliminate the need for human expertise; rather, it relies upon a rigorous upfront investment in operational definitions, expert calibration, and quality assurance to safely scale assessment across larger populations.

### Ethical Implementation and Clinical Safeguards

While LLM-based ratings achieve strong psychometric properties, responsible translation from research to clinical or programmatic use requires stringent ethical, practical, and technical safeguards. The AI rating system validated here is explicitly intended to augment, rather than replace, human clinical judgment. In clinical implementation, it should operate within a human-in-the-loop model as a scalable screening tool, facilitating timely referrals while licensed clinicians retain full decision-making authority. AI-generated scores must never serve as a sole basis for clinical diagnoses, benefits determinations, or access to services, and implementation must include strict oversight to prevent repurposing for punitive or exclusionary surveillance. Furthermore, processing sensitive veteran transcripts necessitates rigorous data privacy protocols, including comprehensive deidentification, enterprise-tier API agreements prohibiting secondary model training, and strict adherence to Health Insurance Portability and Accountability Act of 1996 and VA data security requirements. Prioritizing locally deployable, open-source models may offer the most robust solution for ensuring data sovereignty within VA and Department of Defense contexts.

Addressing the “black box” nature of LLMs is equally critical for clinical acceptability. To ensure transparency, the AI was required to generate a written rationale for each numerical rating, demonstrating that the model attended to substantively relevant narrative content (eg, employment difficulties and identity loss) rather than arbitrary textual features. Additionally, while our partial correlation analyses suggest that convergent validity associations remain stable across age and gender, LLMs may encode societal biases. Future large-scale deployments must include ongoing bias monitoring, formal fairness audits across demographic subgroups, and disaggregated performance metrics to ensure equitable assessment across racial, ethnic, and gender lines.

### Limitations and Future Directions

Several limitations warrant consideration. First, the cross-sectional design provides concurrent validation but precludes establishing predictive validity. Prospective longitudinal studies are necessary to determine whether AI-rated transition stress can accurately predict future clinical outcomes, such as the onset of PTSD or employment difficulties, before symptoms fully manifest. Second, because LLMs are inherently stochastic, minor fluctuations in ratings are possible. Although a temperature setting of 0 (greedy decoding) was utilized to minimize variability, future research must formally evaluate test-retest reliability across repeated runs to quantify stochastic variation. Third, the present findings are specific to the Gemini 2.5 Flash model. Because LLMs undergo continuous updates, “model drift” presents a unique challenge to longitudinal measurement. Our goal here was to determine LLM’s general capabilities in psychometric tasks using unstructured narratives. Future research and clinical deployments must adopt strict model versioning practices and conduct periodic revalidation. Moreover, we did not compare other model’s performance (eg, OpenAI, Anthropic, etc) as our intent was not to determine the best-in-class model or compare performance. Fourth, while the AI-generated themes align closely with established qualitative literature, future studies should incorporate independent human qualitative coding for triangulation to definitively confirm that emergent themes reflect genuine narrative patterns rather than artifacts of the model’s training data.

Regarding sample-related limitations, although we recruited a diverse sample, recruitment through online forums may have introduced sampling bias by excluding veterans who do not use social media. In addition, because the sample consisted exclusively of post-9/11 veterans, further research is needed to determine whether these findings generalize to veterans from earlier service eras or peacetime service. Additionally, while the narrative patterns observed in the low transition stress group—agency, coherence, and redemptive framing—are consistent with theoretical accounts of adaptive functioning, this cross-sectional design does not permit equating narrative style with resilience as a psychological process or outcome. Coherent, future-oriented narratives may reflect genuine psychological adaptation, but they may also be shaped by verbal fluency, self-presentational tendencies, or storytelling ability that operate independently of psychological well-being. Future research using longitudinal designs and multimethod assessment is needed to disentangle narrative coherence from underlying resilience processes. Finally, as the sample was predominantly male, further research is needed to examine whether the thematic associations observed here are consistent among female veterans.

### Conclusion

Couched within these limitations, this study demonstrates that Generative AI, applied with psychometric rigor, can significantly advance our substantive understanding of complex psychological constructs, such as veteran transition stress, while validating a powerful new research methodology. By revealing the distinct architectures (defining narrative arcs and themes) of low and high transition stress, this approach provides a more granular picture of the veteran experience and offers a transformative paradigm for integrating quantitative and qualitative analyses in mental health research.

## Supplementary material

10.2196/90155 Multimedia Appendix 1 Examples of narrative arc for low and high transition stress veterans.

10.2196/90155 Multimedia Appendix 2 Gender differences in transition stress and key themes.

10.2196/90155 Multimedia Appendix 3 Complete prompts for all 5 conditions.

## Abbreviations

AI: artificial intelligence
API: application programming interface
DSM-5: Diagnostic and Statistical Manual of Mental Disorders, Fifth Edition
FDR: false discovery rate
LLM: large language model
LOTTS: loss, trauma, and transition stress
M2CQ: Military to Civilian Questionnaire
PTSD: posttraumatic stress disorder
VA: Veterans Administration
