# Supplementary material for: Use of a Large Language Model to Reveal Narrative Architectures of Veteran Transition Stress: Development and Validation Study
Source: JMIR Ment Health. 2026 Apr 30;13:e90155. doi: 10.2196/90155 (PMC13132017; doi:10.2196/90155)
Supplement: Multimedia Appendix 1 [file mental-v13-e90155-s001.docx]

**Appendix 1. Examples of narrative arc for low and high transition stress veterans**.

**1. Low Transition Stress: Redemptive & Coherent Arcs**

These narratives demonstrate a clear, logical progression. The veterans acknowledge the shift but frame their journey as a forward-moving sequence of planning, executing, and stepping into a new, positive chapter.

**Veteran Example 1:**

*"My experience has been fairly good. I began my transition five years ago now in 20xx. And I went to school, to college, did that for four years. I’ve managed to, because of my service, find programs to help me align, better my academics towards career goals, sort of speak... in a better way to what I did... and what I was capable of."*

- **AI explanation:** This is a perfectly linear, highly coherent narrative. It establishes a clear timeline, a logical sequence of actions (went to college for four years), and a redemptive conclusion (using military experience to successfully align with civilian career goals).

**Veteran Example 2:**

*"My transition I think was probably better than most... my wife is not particularly invested in military life and so I feel like it was a pretty clean break, we moved away and I enrolled in college really quickly. I think I was still actually on terminal leave when I started and started school and treated it like a job and it went really well."*

- **AI explanation:** This narrative arc shows swift, decisive forward momentum. The transition is not framed as a loss, but as a deliberate pivot. The cause-and-effect is clear: leaving the military > moving away > starting school immediately > achieving success.

**Veteran Example 3:**

*"I did a pretty good job of leaning into it, planning for it, you know, kind of getting things I needed to get done. You know, started almost a year out. So, you know, moving my stuff, medical stuff, you know, applying for jobs, applying for school... all that went fairly well. It was kind of a headache at times as government stuff [is]..."*

- **AI explanation:** This perfectly illustrates a coherent narrative structure. The veteran acknowledges friction ("a headache at times"), but the overarching story is one of mastery over the timeline. The plot is driven entirely by the veteran's proactive checklist, resulting in a stable transition

**2. High Transition Stress: Contaminated & Fragmented Arcs**

These narratives lack a logical, forward-moving flow. Instead, they are characterized by derailment. A traumatic event, an institutional failure, or a sudden crisis abruptly alters the trajectory, "spoiling" the veteran's plans and leaving them stuck in a chaotic or unresolved state.

**Veteran Example 1:**

*"It was probably the worst experience of my life. Even topping that of the, my deployment because when I got out of the military, as I mentioned I got injured they told me to change my job or get out so they processed the paperwork... but my ETS date was up and I didn't want to be anything other than infantry and I was still suffering from XXX when, when I ETSed out but they still cleared me so..."*

- **AI explanation:** This is a textbook "Contamination" arc. The veteran's desired trajectory and core identity (being infantry) are abruptly ruined by an injury and perceived institutional indifference. The exit process itself becomes a new trauma ("the worst experience of my life"), tainting the end of their service and leaving them unresolved and physically/psychologically stuck.

**Veteran Example 2:**

*"It was an abrupt change from active duty to civilian life. I was diagnosed with XXX in 20XX and I was an infantry officer about to make lieutenant colonel... so I was forced to retire medically in 20XX.. and then at that same time that that happened my ex-wife asked for a divorce."*

- **AI explanation:** This narrative represents severe fragmentation. The veteran's expected life trajectory (making Lieutenant Colonel) is suddenly derailed by an uncontrollable medical diagnosis, which then immediately compounds with a divorce. The story is an uncontrolled downward spiral dictated entirely by external forces.

**Veteran Example 3:**

*"Get into it was a f***ing rollercoaster I think towards the end of my service I was very numb and once I thought I'd take my life and it was ten times worse when I got out. I was going to get a six-figure job with contracting company or go to school then my XXXX accused me of XXXX, pressing charges on me, wanted me to spend my life in prison."*

- **AI explanation:** This is highly fragmented and chaotic. The narrative jumps from severe internal distress (numbness and suicidal ideation) to a sudden, bizarre external crisis (false XXXX charges) that instantly destroys a promising civilian opportunity. The arc is whiplash-inducing, reflecting a life in freefall without a clear path forward.
