# Supplementary material for: Use of a Large Language Model to Reveal Narrative Architectures of Veteran Transition Stress: Development and Validation Study
Source: JMIR Ment Health. 2026 Apr 30;13:e90155. doi: 10.2196/90155 (PMC13132017; doi:10.2196/90155)
Supplement: Multimedia Appendix 2 [file mental-v13-e90155-s002.docx]

**Appendix 2. Gender Differences in Transition Stress and Key Themes**

| **Aspect** | **Male Veterans** | **Female Veterans (Note: Small sample size, n=20)** |
| --- | --- | --- |
| **Prevalence of High Stress** | High distress is common but distributed across a wide range of causal factors (combat, injury, identity loss, etc.). | Disproportionately represented in the high-distress group. Narratives of severe distress are common. |
| **Primary Driver of Severe Trauma Narratives** | Combat Exposure & Aftermath: Narratives of high trauma most frequently center on intense, direct combat, IEDs, witnessing the death of comrades, and the subsequent survivor's guilt or PTSD. | Military Sexual Trauma (MST) & Institutional Betrayal: The most potent and defining PTE for high-stress female veterans. The trauma of the assault is compounded by a profound sense of betrayal by leadership and the institution. |
| **Illustrative High-Stress Quote Theme** | "My first deployment was Iraq… I think we lost like fifteen guys… in front of my eyes… as far as PTSD goes, that deployment really weighs on me the most." | "I got out because of military sexual trauma… I basically agreed with my commander and helped them cover up what happened to me as long as they let me out honorably… and I feel that, that regret to this day." |
