# Supplementary material for: Use of a Large Language Model to Reveal Narrative Architectures of Veteran Transition Stress: Development and Validation Study
Source: JMIR Ment Health. 2026 Apr 30;13:e90155. doi: 10.2196/90155 (PMC13132017; doi:10.2196/90155)
Supplement: Multimedia Appendix 3 [file mental-v13-e90155-s003.docx]

**Zero Shot**

You are a veteran transition stress rater. Based on the following definition of transition stress here: "Transition stress refers to the psychological and emotional strain experienced during periods of significant change or transition in life. For veterans, these changes may include moving to a new location, finding work and adjusting to civilian employment, reacclimating to life with their spouse, children or other relatives, relationships with non-military friends, managing finances, and managing civilian time and schedule. Transition stress arises from the uncertainty, adjustment, and adaptation required to navigate these changes successfully. It can manifest as anxiety, depression, irritability, difficulty concentrating, sleep disturbances, or other symptoms of stress." And the rating scale here: "1 = Minimal or no distress from transition-related challenges

2 = Minimal to moderate distress due to transition-related challenges

3 =Moderate distress due to transition-related challenges

4 = Moderate to high distress due to transition-related challenges

5 = HIgh distress due to transition-related challenges "

Please rate the level of transition stress in the attached transcripts in the "Trascription_1to20" excelsheet according to the definition and rating scales above. Please provide ratings for all 20 transcriptions and provide explanations for your ratings for each one.

**One shot**

You are a veteran transition stress rater. Based on the following definition of transition stress here: "Transition stress refers to the psychological and emotional strain experienced during periods of significant change or transition in life. For veterans, these changes may include moving to a new location, finding work and adjusting to civilian employment, reacclimating to life with their spouse, children or other relatives, relationships with non-military friends, managing finances, and managing civilian time and schedule. Transition stress arises from the uncertainty, adjustment, and adaptation required to navigate these changes successfully. It can manifest as anxiety, depression, irritability, difficulty concentrating, sleep disturbances, or other symptoms of stress."

And use the rating scale here: "1 = Minimal or no distress from transition-related challenges

2 = Minimal to moderate distress due to transition-related challenges

3 =Moderate distress due to transition-related challenges

4 = Moderate to high distress due to transition-related challenges

5 = High distress due to transition-related challenges "

Please rate the level of transition stress in the attached transcripts in the "Trascription_singleshot" excel sheet according to the definition and rating scales above.

I also attached one example of rating and explanations from expert raters and a few examples of expert perspectives on why this transcript represents this rating. Here is the transcript:

“how they rated transition stress for one transcript, learn how the experts rated transition stress, and provide ratings for all 19 transcriptions and provide explanations for your ratings.

Example Transcript (ID 3007):

“I'll say pretty horrible. It's been pretty horrible. I got out because of military sexual trauma, I got sexually assaulted literally from my recruiter until, and sexual harassment, sexual harassment sexual assault starting with my recruiter all the way until the general or the commander of my unit and like the last day my one of my lasting memories of leaving the military was a bunch of female sergeants surrounding me and screaming at me and saying don't leave don't leave, we need women like you in the military, you're one of the few that stuck up for yourself, you stuck up for other women, you're not taking this shit like we need women like you and I wish that I had them throughout my service but I only had these women like the last two months of my service and I remember just saying no I'm done, I'm mentally and emotionally done, I want out and they kind of just, you know they couldn't fault me and one of them said can't fault you but, there was literally like 10 years ago and I regret getting out, I see women that are getting killed in the military like there's girl Vanessa. Other females that are serving the military and you know they're getting killed by other military personnel and I constantly, and I know this isn't like, doesn't make sense but I'm just like oh if I had stayed would she be alive, if I had stayed would have I made a difference, I always cry when I see one of em. When I got out the military I was so scared of being a statistic, of being a female that turned homeless and crazy or whatever they would say so I started dating this guy and he was my first full blown abusive relationship and it was a nightmare it was an absolute nightmare. It made getting out of the military more stressful because I thought I was, I'm, okay I'm getting out of sexual harassment, I'm getting out of sexual assault, I'm getting away from all this abuse and stuff like that and I walked right into another abusive situation and I couldn't take it, I could not, I mean this guy, the end of the relationship ended with him pulling a gun on me and saying you're not going anywhere and I just left the house I was like you know what, if, if he's going to shoot me, I should turn my back so he shoots me in the back so it shows that you know whatever he was planning to do wasn't, it wasn't self-defense. Another issue that I had after I got out of the military is realizing how much I changed. How much my personality had changed and I hadn't gone back home, I'm originally from Cleveland, Ohio, and I kept all my friends from there and nothing changed, nothing changed with them, I found myself to be more reserved, I found myself to be more thoughtful. I got diagnosed with PTSD but I didn't fully understand it, so I was having and exhibiting you know symptoms of PTSD but I just didn't think anything of it. I still pushed to accomplish a lot of things in my life so after the military, after I got away from the abusive boyfriend I started working full-time, I finished my bachelor's, I got my master's then I went to the peace corps, did a year in the peace corps and the peace corps basically re-ignited all the things I had avoided in the military. I got sexually assaulted there, I got sexually harassed constantly, it was it was literally like, that was like a deployment without a gun. That was like a full-blown deployment. And then I came back after a year and I realized, I'm like legit fucked up. Like in 2018, I was like I'm legit fucked up, I never truly dealt with whatever happened to me in the military, I kept repeating the same thing over and over and over uh finding myself in abusive relationships, abusive friendships, boyfriends, jobs, my jobs were always similarly abusive, I have felt attached to working to the government I felt like there's, no, I have to I have to work for the government I have to work for the government cause it's all I know. So I got out of the peace corps, moved to DC, and now I find myself in the same exact situation as the military where I got raped by a co-worker, I got harassed all the time, I tried to report it, nobody is helping me and I finally just completely lost complete I just lost complete control and now I'm trying to fix myself, I'm trying not to repeat what I did in the military, that I just stay silent, like in order for me to get out of the military there you know, they did an investigation, it came back oh you're not lying, you, we thought you were lying but you're not lying, okay what can we do to keep you quiet and I basically agreed with my commander and help them cover up what happened to me as long as they let me out honorably and gave me my benefits, and I feel that, that regret to this day, when I went to the peace corps bad things happened to me, I just never told anyone. I just said fuck it and I never told anyone, and now at my job, which is a government agency I just full-blown exploded, I didn't keep quiet, I said I'm not going do what I did before, I sent out mass emails and said what was happening, I spoke openly about stuff, I got harassed more, I refused to quit, like I fought, they tried to wrongfully terminate me at least 3 separate times and I was able to provide evidence that they were lying and outside of that I am full blown trying to do counselling. No, I'm not walking away from counselling, I'm doing everything that I can do to better myself. And moving forward in the future, I don't think I'm going to work for the government anymore. I don't think, I thought all these years I thought the military was the fucked up one but it's not it's the entire government it's the entire structure of the government. I'm sick of getting raped honestly, I don't know how else to say that but I'm sick of getting raped, I'm sick of keeping quiet, I'm sick of this lifestyle and my thought is this time, instead of running away from the issue, I stayed at the job, I'm trying to get help, you know, counselling and stuff like that but I know that looking forward in the future I'm going to change my life and do everything that I wanted to do that I'm scared to do, like I really don't want to work for the government, I'm not a money-hungry person, I am really nice person, and I always get treated badly, and my job is because I had no boundaries. And I believed what they told me in the military, you're ugly, you're stupid, your family didn't want you that's why you're here and it affected my self-esteem and, I just don't want to keep repeating, I just don't want to keep repeating it.”

Expert Rating: 4.9/5 (average of 10 expert raters)

Expert Explanation 1 (rater 5): The participant has experienced repeated sexual abuse both during her service and during transition. She blamed herself for getting out of the military. She was in an abusive relationship. She has been through a lot

Expert Explanation 2 (rater 7): This one is self-explanatory; this individual experience severe distress and trauma as well as a PTSD diagnosis; the stress impacted multiple life domains, over an extended period of time; definitely a 5

Expert Explanation 3 (rater 11): Seemed very distressed due to sexual assault and abusive relationship. They cannot relate to their friends from before the military and were diagnosed with PTSD.

**Two Shots**

Prompts: You are a veteran transition stress rater. Based on the following definition of transition stress here: "Transition stress refers to the psychological and emotional strain experienced during periods of significant change or transition in life. For veterans, these changes may include moving to a new location, finding work and adjusting to civilian employment, reacclimating to life with their spouse, children or other relatives, relationships with non-military friends, managing finances, and managing civilian time and schedule. Transition stress arises from the uncertainty, adjustment, and adaptation required to navigate these changes successfully. It can manifest as anxiety, depression, irritability, difficulty concentrating, sleep disturbances, or other symptoms of stress."

And use the rating scale here: "1 = Minimal or no distress from transition-related challenges

2 = Minimal to moderate distress due to transition-related challenges

3 =Moderate distress due to transition-related challenges

4 = Moderate to high distress due to transition-related challenges

5 = High distress due to transition-related challenges "

Please rate the level of transition stress in the attached transcripts in the "Trascription_without3007&3030" excel sheet according to the definition and rating scales above.

I also attached two transcripts of ratings and explanations from expert raters and a few examples of expert perspectives on why those two transcripts represent these ratings. Here are the transcripts:

Learn how the experts rated transition stress , and provide ratings for all 18 transcriptions and provide explanations for your ratings.

Example One Transcript (ID 3007):

“I'll say pretty horrible. It's been pretty horrible. I got out because of military sexual trauma, I got sexually assaulted literally from my recruiter until, and sexual harassment, sexual harassment sexual assault starting with my recruiter all the way until the general or the commander of my unit and like the last day my one of my lasting memories of leaving the military was a bunch of female sergeants surrounding me and screaming at me and saying don't leave don't leave, we need women like you in the military, you're one of the few that stuck up for yourself, you stuck up for other women, you're not taking this shit like we need women like you and I wish that I had them throughout my service but I only had these women like the last two months of my service and I remember just saying no I'm done, I'm mentally and emotionally done, I want out and they kind of just, you know they couldn't fault me and one of them said can't fault you but, there was literally like 10 years ago and I regret getting out, I see women that are getting killed in the military like there's girl Vanessa. Other females that are serving the military and you know they're getting killed by other military personnel and I constantly, and I know this isn't like, doesn't make sense but I'm just like oh if I had stayed would she be alive, if I had stayed would have I made a difference, I always cry when I see one of em. When I got out the military I was so scared of being a statistic, of being a female that turned homeless and crazy or whatever they would say so I started dating this guy and he was my first full blown abusive relationship and it was a nightmare it was an absolute nightmare. It made getting out of the military more stressful because I thought I was, I'm, okay I'm getting out of sexual harassment, I'm getting out of sexual assault, I'm getting away from all this abuse and stuff like that and I walked right into another abusive situation and I couldn't take it, I could not, I mean this guy, the end of the relationship ended with him pulling a gun on me and saying you're not going anywhere and I just left the house I was like you know what, if, if he's going to shoot me, I should turn my back so he shoots me in the back so it shows that you know whatever he was planning to do wasn't, it wasn't self-defense. Another issue that I had after I got out of the military is realizing how much I changed. How much my personality had changed and I hadn't gone back home, I'm originally from Cleveland, Ohio, and I kept all my friends from there and nothing changed, nothing changed with them, I found myself to be more reserved, I found myself to be more thoughtful. I got diagnosed with PTSD but I didn't fully understand it, so I was having and exhibiting you know symptoms of PTSD but I just didn't think anything of it. I still pushed to accomplish a lot of things in my life so after the military, after I got away from the abusive boyfriend I started working full-time, I finished my bachelor's, I got my master's then I went to the peace corps, did a year in the peace corps and the peace corps basically re-ignited all the things I had avoided in the military. I got sexually assaulted there, I got sexually harassed constantly, it was it was literally like, that was like a deployment without a gun. That was like a full-blown deployment. And then I came back after a year and I realized, I'm like legit fucked up. Like in 2018, I was like I'm legit fucked up, I never truly dealt with whatever happened to me in the military, I kept repeating the same thing over and over and over uh finding myself in abusive relationships, abusive friendships, boyfriends, jobs, my jobs were always similarly abusive, I have felt attached to working to the government I felt like there's, no, I have to I have to work for the government I have to work for the government cause it's all I know. So I got out of the peace corps, moved to DC, and now I find myself in the same exact situation as the military where I got raped by a co-worker, I got harassed all the time, I tried to report it, nobody is helping me and I finally just completely lost complete I just lost complete control and now I'm trying to fix myself, I'm trying not to repeat what I did in the military, that I just stay silent, like in order for me to get out of the military there you know, they did an investigation, it came back oh you're not lying, you, we thought you were lying but you're not lying, okay what can we do to keep you quiet and I basically agreed with my commander and help them cover up what happened to me as long as they let me out honorably and gave me my benefits, and I feel that, that regret to this day, when I went to the peace corps bad things happened to me, I just never told anyone. I just said fuck it and I never told anyone, and now at my job, which is a government agency I just full-blown exploded, I didn't keep quiet, I said I'm not going do what I did before, I sent out mass emails and said what was happening, I spoke openly about stuff, I got harassed more, I refused to quit, like I fought, they tried to wrongfully terminate me at least 3 separate times and I was able to provide evidence that they were lying and outside of that I am full blown trying to do counselling. No, I'm not walking away from counselling, I'm doing everything that I can do to better myself. And moving forward in the future, I don't think I'm going to work for the government anymore. I don't think, I thought all these years I thought the military was the fucked up one but it's not it's the entire government it's the entire structure of the government. I'm sick of getting raped honestly, I don't know how else to say that but I'm sick of getting raped, I'm sick of keeping quiet, I'm sick of this lifestyle and my thought is this time, instead of running away from the issue, I stayed at the job, I'm trying to get help, you know, counselling and stuff like that but I know that looking forward in the future I'm going to change my life and do everything that I wanted to do that I'm scared to do, like I really don't want to work for the government, I'm not a money-hungry person, I am really nice person, and I always get treated badly, and my job is because I had no boundaries. And I believed what they told me in the military, you're ugly, you're stupid, your family didn't want you that's why you're here and it affected my self-esteem and, I just don't want to keep repeating, I just don't want to keep repeating it.”

Expert Rating: 4.9/5 (average of 10 expert raters)

Expert Explanation 1 (rater 5): The participant has experienced repeated sexual abuse both during her service and during transition. She blamed herself for getting out of the military. She was in an abusive relationship. She has been through a lot

Expert Explanation 2 (rater 7): This one is self-explanatory; this individual experience severe distress and trauma as well as a PTSD diagnosis; the stress impacted multiple life domains, over an extended period of time; definitely a 5

Expert Explanation 3 (rater 11): Seemed very distressed due to sexual assault and abusive relationship. They cannot relate to their friends from before the military and were diagnosed with PTSD.

Example Two Transcript (ID 3030):

my transition I think was probably better than most I was married when I transitioned out, am still married, and my my wife is not particularly invested in in military life and so I feel like it was a pretty clean break, we moved away and I enrolled in college really quickly well I think I was still actually on terminal leave when I started and started school and treated it like a job and it went really well and I don't think that I have really had any any hiccups since getting out I don't really know that I have too much to add it's been pretty uneventful.

Expert Rating: 1/5 (average of 10 expert raters)

Expert Explanation 1 (rater 5): Describes the transition as well and uneventful.

Expert Explanation 2 (rater 7): This feels like a 1; this individual mentions a "clean break" from the military, no mention of distress, "no hiccups."

Expert Explanation 3 (rater 11): Expressed no distress

**Three shots**

You are a veteran transition stress rater. Based on the following definition of transition stress here: "Transition stress refers to the psychological and emotional strain experienced during periods of significant change or transition in life. For veterans, these changes may include moving to a new location, finding work and adjusting to civilian employment, reacclimating to life with their spouse, children or other relatives, relationships with non-military friends, managing finances, and managing civilian time and schedule. Transition stress arises from the uncertainty, adjustment, and adaptation required to navigate these changes successfully. It can manifest as anxiety, depression, irritability, difficulty concentrating, sleep disturbances, or other symptoms of stress."

And use the rating scale here: "1 = Minimal or no distress from transition-related challenges

2 = Minimal to moderate distress due to transition-related challenges

3 =Moderate distress due to transition-related challenges

4 = Moderate to high distress due to transition-related challenges

5 = High distress due to transition-related challenges "

Please rate the level of transition stress in the attached transcripts in the "Trascription_without3007&3030&3036" excel sheet according to the definition and rating scales above.

I also attached three transcripts of ratings and explanations from expert raters and a few examples of expert perspectives on why those three transcripts represent these ratings. Here are the transcripts:

Learn how the experts rated transition stress , and provide ratings for all 17 transcriptions and provide explanations for your ratings.

Example One Transcript (ID 3007):

“I'll say pretty horrible. It's been pretty horrible. I got out because of military sexual trauma, I got sexually assaulted literally from my recruiter until, and sexual harassment, sexual harassment sexual assault starting with my recruiter all the way until the general or the commander of my unit and like the last day my one of my lasting memories of leaving the military was a bunch of female sergeants surrounding me and screaming at me and saying don't leave don't leave, we need women like you in the military, you're one of the few that stuck up for yourself, you stuck up for other women, you're not taking this shit like we need women like you and I wish that I had them throughout my service but I only had these women like the last two months of my service and I remember just saying no I'm done, I'm mentally and emotionally done, I want out and they kind of just, you know they couldn't fault me and one of them said can't fault you but, there was literally like 10 years ago and I regret getting out, I see women that are getting killed in the military like there's girl Vanessa. Other females that are serving the military and you know they're getting killed by other military personnel and I constantly, and I know this isn't like, doesn't make sense but I'm just like oh if I had stayed would she be alive, if I had stayed would have I made a difference, I always cry when I see one of em. When I got out the military I was so scared of being a statistic, of being a female that turned homeless and crazy or whatever they would say so I started dating this guy and he was my first full blown abusive relationship and it was a nightmare it was an absolute nightmare. It made getting out of the military more stressful because I thought I was, I'm, okay I'm getting out of sexual harassment, I'm getting out of sexual assault, I'm getting away from all this abuse and stuff like that and I walked right into another abusive situation and I couldn't take it, I could not, I mean this guy, the end of the relationship ended with him pulling a gun on me and saying you're not going anywhere and I just left the house I was like you know what, if, if he's going to shoot me, I should turn my back so he shoots me in the back so it shows that you know whatever he was planning to do wasn't, it wasn't self-defense. Another issue that I had after I got out of the military is realizing how much I changed. How much my personality had changed and I hadn't gone back home, I'm originally from Cleveland, Ohio, and I kept all my friends from there and nothing changed, nothing changed with them, I found myself to be more reserved, I found myself to be more thoughtful. I got diagnosed with PTSD but I didn't fully understand it, so I was having and exhibiting you know symptoms of PTSD but I just didn't think anything of it. I still pushed to accomplish a lot of things in my life so after the military, after I got away from the abusive boyfriend I started working full-time, I finished my bachelor's, I got my master's then I went to the peace corps, did a year in the peace corps and the peace corps basically re-ignited all the things I had avoided in the military. I got sexually assaulted there, I got sexually harassed constantly, it was it was literally like, that was like a deployment without a gun. That was like a full-blown deployment. And then I came back after a year and I realized, I'm like legit fucked up. Like in 2018, I was like I'm legit fucked up, I never truly dealt with whatever happened to me in the military, I kept repeating the same thing over and over and over uh finding myself in abusive relationships, abusive friendships, boyfriends, jobs, my jobs were always similarly abusive, I have felt attached to working to the government I felt like there's, no, I have to I have to work for the government I have to work for the government cause it's all I know. So I got out of the peace corps, moved to DC, and now I find myself in the same exact situation as the military where I got raped by a co-worker, I got harassed all the time, I tried to report it, nobody is helping me and I finally just completely lost complete I just lost complete control and now I'm trying to fix myself, I'm trying not to repeat what I did in the military, that I just stay silent, like in order for me to get out of the military there you know, they did an investigation, it came back oh you're not lying, you, we thought you were lying but you're not lying, okay what can we do to keep you quiet and I basically agreed with my commander and help them cover up what happened to me as long as they let me out honorably and gave me my benefits, and I feel that, that regret to this day, when I went to the peace corps bad things happened to me, I just never told anyone. I just said fuck it and I never told anyone, and now at my job, which is a government agency I just full-blown exploded, I didn't keep quiet, I said I'm not going do what I did before, I sent out mass emails and said what was happening, I spoke openly about stuff, I got harassed more, I refused to quit, like I fought, they tried to wrongfully terminate me at least 3 separate times and I was able to provide evidence that they were lying and outside of that I am full blown trying to do counselling. No, I'm not walking away from counselling, I'm doing everything that I can do to better myself. And moving forward in the future, I don't think I'm going to work for the government anymore. I don't think, I thought all these years I thought the military was the fucked up one but it's not it's the entire government it's the entire structure of the government. I'm sick of getting raped honestly, I don't know how else to say that but I'm sick of getting raped, I'm sick of keeping quiet, I'm sick of this lifestyle and my thought is this time, instead of running away from the issue, I stayed at the job, I'm trying to get help, you know, counselling and stuff like that but I know that looking forward in the future I'm going to change my life and do everything that I wanted to do that I'm scared to do, like I really don't want to work for the government, I'm not a money-hungry person, I am really nice person, and I always get treated badly, and my job is because I had no boundaries. And I believed what they told me in the military, you're ugly, you're stupid, your family didn't want you that's why you're here and it affected my self-esteem and, I just don't want to keep repeating, I just don't want to keep repeating it.”

Expert Rating: 4.9/5 (average of 10 expert raters)

Expert Explanation 1 (rater 5): The participant has experienced repeated sexual abuse both during her service and during transition. She blamed herself for getting out of the military. She was in an abusive relationship. She has been through a lot

Expert Explanation 2 (rater 7): This one is self-explanatory; this individual experience severe distress and trauma as well as a PTSD diagnosis; the stress impacted multiple life domains, over an extended period of time; definitely a 5

Expert Explanation 3 (rater 11): Seemed very distressed due to sexual assault and abusive relationship. They cannot relate to their friends from before the military and were diagnosed with PTSD.

Example Two Transcript (ID 3030):

my transition I think was probably better than most I was married when I transitioned out, am still married, and my my wife is not particularly invested in in military life and so I feel like it was a pretty clean break, we moved away and I enrolled in college really quickly well I think I was still actually on terminal leave when I started and started school and treated it like a job and it went really well and I don't think that I have really had any any hiccups since getting out I don't really know that I have too much to add it's been pretty uneventful.

Expert Rating: 1/5 (average of 10 expert raters)

Expert Explanation 1 (rater 5): Describes the transition as well and uneventful.

Expert Explanation 2 (rater 7): This feels like a 1; this individual mentions a "clean break" from the military, no mention of distress, "no hiccups."

Expert Explanation 3 (rater 11): Expressed no distress

Example Three Transcript (ID 3036):

It was an abrupt change from active duty to civilian life. I was diagnosed with MS in 2017 and I was an infantry officer about to make lieutenant colonel you can't continue because you're not deployable so I was forced to retire medically in 2019. I still did 20 years of service it was great and then at that same time that that happened my ex-wife asked for a divorce and so going through you know medically, getting a divorced all that stuff really shaped my transition in a pretty negative way. I had a year and a half to transition. I applied for 119 jobs. I had like I think 96 versions of my resume, I had 74 distinct cover letters and out of that I got 3 interviews. I mean I have a master's degree from Columbia in organizational psychology and so I thought that I would be able to find meaningful work that really made me want to work and like do good and two of those interviews I walked out of because they were just laughable. It's clear that they didn't know who was in front of them. They were talking to me about behavioral scales and stuff like that and didn't realize that Warner Berk was one of my instructors and I could dissect these things like crazy, asking me dumb questions, questions that were illegal to ask me about like what I'm making in retirement and so it was a very negative experience and I was super fortunate to fall into this job that I have now. I run a youth soccer club in Nashville, so I go from you know infantry officer to now I'm running a youth soccer club it was kind of a shock in the system. You know you can't do the same things in dealing with customers, you know parents, as you could in the military although I would like to with a few of them and it's been rewarding in the sense that my kids live in Arizona with their mother and they, it fills a void for me because my kids aren't around me every day and so it's, I love going to the field and seeing the kids and watching them play and have fun and learn, learn about the game and things like that but I cannot stand dealing with people at all. I used to be an extravert, and then in this transition my personality has changed, and I have found that I am because of the transition and because of the difficulty with the transition I am very much an introvert and don't want to interact with people which is a big change for me. So it was, it was really difficult, it's still difficult as I'm still trying to find a career and not a job so it's been close to you know 4, going on 5 years of me trying to find a career and not just this job and so it continues to be difficult, it's frustrating, its stressful. One thing that helps me is I help you know other friends of mine that are retiring. I'm very vocal with them and help them and try to do my best to help other people. No, I'm, I mean just that its, its I'm really tired of the stigma that comes with being in the army and when it comes to corporations and things like that, I'm really tired of it. I'm not, I'm not broken, I'm not more broken than anybody else, right.

Expert Rating: 3.8/5 (average of 10 expert raters)

Expert Explanation 1 (rater 5): Went through serious medical problems and a divorce. Had problems trying to find a job. Is content with current job but still looking for a career. Misses their children and feel the stigma of being a veteran.

Expert Explanation 2 (rater 7): This individual mentions multiple domains; marriage, medical, employment. This is definitely a 3, maybe a 4. not super high emotional valence but definitely talks about stress; mentions "frustration," "stress" and "stigma" - maybe a 4...

Expert Explanation 3 (rater 11): expressed distress in several life domains: career, health, and relationships

**Five Shots**

You are a veteran transition stress rater. Based on the following definition of transition stress here: "Transition stress refers to the psychological and emotional strain experienced during periods of significant change or transition in life. For veterans, these changes may include moving to a new location, finding work and adjusting to civilian employment, reacclimating to life with their spouse, children or other relatives, relationships with non-military friends, managing finances, and managing civilian time and schedule. Transition stress arises from the uncertainty, adjustment, and adaptation required to navigate these changes successfully. It can manifest as anxiety, depression, irritability, difficulty concentrating, sleep disturbances, or other symptoms of stress."

And use the rating scale here: "1 = Minimal or no distress from transition-related challenges

2 = Minimal to moderate distress due to transition-related challenges

3 =Moderate distress due to transition-related challenges

4 = Moderate to high distress due to transition-related challenges

5 = High distress due to transition-related challenges "

Please rate the level of transition stress in the attached transcripts in the "Trascription_without3030&3017&3033&3036&3007" excel sheet according to the definition and rating scales above.

I also attached five transcripts of ratings and explanations from expert raters and a few examples of expert perspectives on why those five transcripts represent these ratings. Here are the transcripts:

Learn how the experts rated transition stress , and provide ratings for all 15 transcriptions and provide explanations for your ratings.

Example One Transcript (ID 3007):

“I'll say pretty horrible. It's been pretty horrible. I got out because of military sexual trauma, I got sexually assaulted literally from my recruiter until, and sexual harassment, sexual harassment sexual assault starting with my recruiter all the way until the general or the commander of my unit and like the last day my one of my lasting memories of leaving the military was a bunch of female sergeants surrounding me and screaming at me and saying don't leave don't leave, we need women like you in the military, you're one of the few that stuck up for yourself, you stuck up for other women, you're not taking this shit like we need women like you and I wish that I had them throughout my service but I only had these women like the last two months of my service and I remember just saying no I'm done, I'm mentally and emotionally done, I want out and they kind of just, you know they couldn't fault me and one of them said can't fault you but, there was literally like 10 years ago and I regret getting out, I see women that are getting killed in the military like there's girl Vanessa. Other females that are serving the military and you know they're getting killed by other military personnel and I constantly, and I know this isn't like, doesn't make sense but I'm just like oh if I had stayed would she be alive, if I had stayed would have I made a difference, I always cry when I see one of em. When I got out the military I was so scared of being a statistic, of being a female that turned homeless and crazy or whatever they would say so I started dating this guy and he was my first full blown abusive relationship and it was a nightmare it was an absolute nightmare. It made getting out of the military more stressful because I thought I was, I'm, okay I'm getting out of sexual harassment, I'm getting out of sexual assault, I'm getting away from all this abuse and stuff like that and I walked right into another abusive situation and I couldn't take it, I could not, I mean this guy, the end of the relationship ended with him pulling a gun on me and saying you're not going anywhere and I just left the house I was like you know what, if, if he's going to shoot me, I should turn my back so he shoots me in the back so it shows that you know whatever he was planning to do wasn't, it wasn't self-defense. Another issue that I had after I got out of the military is realizing how much I changed. How much my personality had changed and I hadn't gone back home, I'm originally from Cleveland, Ohio, and I kept all my friends from there and nothing changed, nothing changed with them, I found myself to be more reserved, I found myself to be more thoughtful. I got diagnosed with PTSD but I didn't fully understand it, so I was having and exhibiting you know symptoms of PTSD but I just didn't think anything of it. I still pushed to accomplish a lot of things in my life so after the military, after I got away from the abusive boyfriend I started working full-time, I finished my bachelor's, I got my master's then I went to the peace corps, did a year in the peace corps and the peace corps basically re-ignited all the things I had avoided in the military. I got sexually assaulted there, I got sexually harassed constantly, it was it was literally like, that was like a deployment without a gun. That was like a full-blown deployment. And then I came back after a year and I realized, I'm like legit fucked up. Like in 2018, I was like I'm legit fucked up, I never truly dealt with whatever happened to me in the military, I kept repeating the same thing over and over and over uh finding myself in abusive relationships, abusive friendships, boyfriends, jobs, my jobs were always similarly abusive, I have felt attached to working to the government I felt like there's, no, I have to I have to work for the government I have to work for the government cause it's all I know. So I got out of the peace corps, moved to DC, and now I find myself in the same exact situation as the military where I got raped by a co-worker, I got harassed all the time, I tried to report it, nobody is helping me and I finally just completely lost complete I just lost complete control and now I'm trying to fix myself, I'm trying not to repeat what I did in the military, that I just stay silent, like in order for me to get out of the military there you know, they did an investigation, it came back oh you're not lying, you, we thought you were lying but you're not lying, okay what can we do to keep you quiet and I basically agreed with my commander and help them cover up what happened to me as long as they let me out honorably and gave me my benefits, and I feel that, that regret to this day, when I went to the peace corps bad things happened to me, I just never told anyone. I just said fuck it and I never told anyone, and now at my job, which is a government agency I just full-blown exploded, I didn't keep quiet, I said I'm not going do what I did before, I sent out mass emails and said what was happening, I spoke openly about stuff, I got harassed more, I refused to quit, like I fought, they tried to wrongfully terminate me at least 3 separate times and I was able to provide evidence that they were lying and outside of that I am full blown trying to do counselling. No, I'm not walking away from counselling, I'm doing everything that I can do to better myself. And moving forward in the future, I don't think I'm going to work for the government anymore. I don't think, I thought all these years I thought the military was the fucked up one but it's not it's the entire government it's the entire structure of the government. I'm sick of getting raped honestly, I don't know how else to say that but I'm sick of getting raped, I'm sick of keeping quiet, I'm sick of this lifestyle and my thought is this time, instead of running away from the issue, I stayed at the job, I'm trying to get help, you know, counselling and stuff like that but I know that looking forward in the future I'm going to change my life and do everything that I wanted to do that I'm scared to do, like I really don't want to work for the government, I'm not a money-hungry person, I am really nice person, and I always get treated badly, and my job is because I had no boundaries. And I believed what they told me in the military, you're ugly, you're stupid, your family didn't want you that's why you're here and it affected my self-esteem and, I just don't want to keep repeating, I just don't want to keep repeating it.”

Expert Rating: 4.9/5 (average of 10 expert raters)

Expert Explanation 1 (rater 5): The participant has experienced repeated sexual abuse both during her service and during transition. She blamed herself for getting out of the military. She was in an abusive relationship. She has been through a lot

Expert Explanation 2 (rater 7): This one is self-explanatory; this individual experience severe distress and trauma as well as a PTSD diagnosis; the stress impacted multiple life domains, over an extended period of time; definitely a 5

Expert Explanation 3 (rater 11): Seemed very distressed due to sexual assault and abusive relationship. They cannot relate to their friends from before the military and were diagnosed with PTSD.

Example Two Transcript (ID 3030):

my transition I think was probably better than most I was married when I transitioned out, am still married, and my my wife is not particularly invested in in military life and so I feel like it was a pretty clean break, we moved away and I enrolled in college really quickly well I think I was still actually on terminal leave when I started and started school and treated it like a job and it went really well and I don't think that I have really had any any hiccups since getting out I don't really know that I have too much to add it's been pretty uneventful.

Expert Rating: 1/5 (average of 10 expert raters)

Expert Explanation 1 (rater 5): Describes the transition as well and uneventful.

Expert Explanation 2 (rater 7): This feels like a 1; this individual mentions a "clean break" from the military, no mention of distress, "no hiccups."

Expert Explanation 3 (rater 11): Expressed no distress

Example Three Transcript (ID 3036):

It was an abrupt change from active duty to civilian life. I was diagnosed with MS in 2017 and I was an infantry officer about to make lieutenant colonel you can't continue because you're not deployable so I was forced to retire medically in 2019. I still did 20 years of service it was great and then at that same time that that happened my ex-wife asked for a divorce and so going through you know medically, getting a divorced all that stuff really shaped my transition in a pretty negative way. I had a year and a half to transition. I applied for 119 jobs. I had like I think 96 versions of my resume, I had 74 distinct cover letters and out of that I got 3 interviews. I mean I have a master's degree from Columbia in organizational psychology and so I thought that I would be able to find meaningful work that really made me want to work and like do good and two of those interviews I walked out of because they were just laughable. It's clear that they didn't know who was in front of them. They were talking to me about behavioral scales and stuff like that and didn't realize that Warner Berk was one of my instructors and I could dissect these things like crazy, asking me dumb questions, questions that were illegal to ask me about like what I'm making in retirement and so it was a very negative experience and I was super fortunate to fall into this job that I have now. I run a youth soccer club in Nashville, so I go from you know infantry officer to now I'm running a youth soccer club it was kind of a shock in the system. You know you can't do the same things in dealing with customers, you know parents, as you could in the military although I would like to with a few of them and it's been rewarding in the sense that my kids live in Arizona with their mother and they, it fills a void for me because my kids aren't around me every day and so it's, I love going to the field and seeing the kids and watching them play and have fun and learn, learn about the game and things like that but I cannot stand dealing with people at all. I used to be an extravert, and then in this transition my personality has changed, and I have found that I am because of the transition and because of the difficulty with the transition I am very much an introvert and don't want to interact with people which is a big change for me. So it was, it was really difficult, it's still difficult as I'm still trying to find a career and not a job so it's been close to you know 4, going on 5 years of me trying to find a career and not just this job and so it continues to be difficult, it's frustrating, its stressful. One thing that helps me is I help you know other friends of mine that are retiring. I'm very vocal with them and help them and try to do my best to help other people. No, I'm, I mean just that its, its I'm really tired of the stigma that comes with being in the army and when it comes to corporations and things like that, I'm really tired of it. I'm not, I'm not broken, I'm not more broken than anybody else, right.

Expert Rating: 3.8/5 (average of 10 expert raters)

Expert Explanation 1 (rater 5): Went through serious medical problems and a divorce. Had problems trying to find a job. Is content with current job but still looking for a career. Misses their children and feel the stigma of being a veteran.

Expert Explanation 2 (rater 7): This individual mentions multiple domains; marriage, medical, employment. This is definitely a 3, maybe a 4. not super high emotional valence but definitely talks about stress; mentions "frustration," "stress" and "stigma" - maybe a 4...

Expert Explanation 3 (rater 11): expressed distress in several life domains: career, health, and relationships

Example Four Transcript (ID 3017):

So, it was a little rough going at first just transitioning from doing, being in the military every day to coming up with the arrangement of like interviewing, finding a job. It took me I think 6 months to actually gain full employment, going through the interview process and everything, so I was effectively unemployed for 6 months, which you know had downs for financial effects, you know, fortunately I'm doing better now, here, 4 or 5 years later. But that was the biggest source of anxiety for me. We had to go through all of our mandatory classes when I was separating, they call it tabs, I don't know what they call it nowadays, but it's like a recon class where you do briefings on the veterans administration, you know resume, job skills, and just yeah I don't know civilian type stuff. I ended up getting a rating with the veteran's administration for 20% due to some job-related issues, which I still receive still. Still a part of the VA. So, throughout all that, I actually transitioned into the reserves, but it was a different beast entirely because you're only doing it for 2 days a month, and, while it was nice to have a little bit of money coming in, while I was effectively unemployed, you know, it's a small percentage of what you were making before. So, on the flip side, I did get to go back home, so I wasn't at the mercy of wherever it is they stationed us, but, you know, ultimately, I think it I feel like it was worth the transition, and I ended up in a better place. I was just tired of being stagnant and stuck in my military job because it was looking like it was going to be 4 more years of doing exactly what I was doing and I was tired of it. Yeah, I think that pretty much encapsulates my transition.

Expert Rating: 2/5 (average of 10 expert raters)

Expert Explanation 1 (rater 5): Had issues regarding employment and maintenance.

Expert Explanation 2 (rater 7): This feels like a 2 as there was mild to moderate distress in one life domain (employment) which impacted another domain (financial). However, the emotional valence seems relatively low.

Expert Explanation 3 (rater 11): They seemed distressed, but only in one domain, which was job-related stress and financial implications from that.

Example Five Transcript (ID 3033):

My experience is a little different, because I was a Reservist for most of the time that I was in the military. So, active duty and reserves. So, I was activated for short periods of time, and so I was in between civilian life and going back into the military being more active duty, going on deployments. So transition, it was actually harder while I was in the military to try to figure out how to be a civilian half, part of the time, than actually once I was fully a civilian. I left the military in 2015, and I decided I wanted to give my hand, you know, try school, use the GI Bill. And so I did really well the first few semesters. I was taking classes that I enjoyed. I was learning different things. I was trying to figure out okay, what am I gonna do with this And it provided me a source of income. Previously leaving the military I was also married and divorced and I have a son. And so, it gave me a lot of time to be with him. And then, couple semesters into it, I lost interest. I wasn't enjoying it. I was having trouble, focusing on, on the work, on going into class, on I just didn't want to be there anymore. I was taking, I had to start taking some more of the classes that I didn't necessarily want to more of the generals and I was having trouble identifying, you know, writing, especially English English has, has plagued me because I it's writing about things that I don't care about, and I couldn't ever find a focused, like, class that would discuss things that I cared about you know, I, I tried to take an English class, and I didn't realize it was like a hip hop kind of class, and you write about all these hip hop artists, and I, it didn't interest me at all. And then I started having family problems, and, me and my ex-wife were having custody issues going back and forth, and so that's pretty typical, you know, divorce in the military but I found my way. I was able to be successful. I found my current wife and, she was really able to help me kind of get on, because I was depressed for a while there. I was unhappy. I didn't know where I was gonna go. I didn't know what direction I was gonna take. And my wife really helped me, figure out and navigate some of that. I eventually got a job with the federal government, and I've switched around a couple different jobs there. But I finally kind of found where I fit in, and I'm in an agency, and my work group is a lot of veterans, not all, but a lot them. And so, we all have a chance to connect and talk and, be able to share those kinds of experiences. And so, while they're not necessarily friends or people that I, I still spend quite a bit of time with them, so they understand me, and we can connect on that level, be jovial and joke about things that are okay for military to joke about and not for regular civilians. And then I've, you know, I used to have a lot more friends. I used to be a lot more gregarious. I'm not anymore. I'm more guarded. I'm, more selective of who I, you know, talk to and who I, let into my inner circle, I guess you could say. So, I have fewer friends, but, I feel like those ones are lasting. I don't know if that's military or just growing up. But I joined when I was 19, and, now I'm, you know, older and so, some of that's I think just growing up. Some of that's, just trying to figure out, you know, life. But I've been really blessed because I've been able to use a lot of my benefits, it's really set me up for life, and it's gonna set my son up. Once I finally got into the VA system, I started, you know, getting, some of the care, so I haven't had a lot of issues. But, I'm not going to VA for all of my care. I'm going for mostly my civilian doctor because I, I do have some, injuries some back pain, some back injuries, some digestive issues and things like that so I have some things that are leftover from the military that are plaguing me, but I don't want to necessarily go to the VA because I'm not, I don't know. VA is very, hit or miss. Sometimes they really help, and sometimes they don't. And, I have a friend who was a VA nurse practitioner, and he talks it up all the time. He lives in Denver now, but, I know that there are good people that work in the VA, but I, just, I've heard so many horror stories that I just don't know if I even want to try and go that route. So, I'm just gonna stick with my civilian doctor for right now, and hopefully that will help. Yeah, I mean the benefits are helpful, but, not everybody uses them I know. A lot of them aren't, and I'm actually gonna be using, some benefits I have already to learn about business. And, I'm gonna try and start a business myself, on the side kind of thing. I don't know if it'll be successful, but I'm willing to give it a try. And then we'll see if my career takes me somewhere else at this point. But it's been nice to be able to connect with other people that share the experiences, especially younger veterans or veterans around my age or those that went to Iraq and things because they understand that kind of aspect. I've talked to older veterans, and not that their experiences are any less, they're just different. And so, a lot of times, like, trying to connect with a Vietnam veteran or a Dessert Storm veteran can be a lot different than trying to connect with someone who was in Iraq or one of the surrounding areas as well.

Expert Rating: 3/5 (average of 10 expert raters)

Expert Explanation 1 (rater 5): It took a while for the participant to find a job they fit in. They have been through a divorce and had custody issues. They didn't feel much engaged during college and is less social compared to before. Yet, they suggest that being in the military, especially being half civilian half solider, more difficult.

Expert Explanation 2 (rater 7): This feels like a 3 because there is moderate stress in multiple life domains; the individual mentions divorce and not enjoying education, as well as physical injuries and personal changes (e.g. being more reserved / introverted and not as gregarious); however, the distress is tempered by positive emotions and a perceieved ability to navigate the challenges; maybe a 4 just based on the challenges themselves, but given the emotional valence of the individual, I'm going with a 3

Expert Explanation 3 (rater 11): Distress in several domains stopped enjoying school, family problems, selective with friends due to disconnect.
